# Recurrent isolated optic neuritis: A study on 22 patients

**Published:** 2017-07-06

**Authors:** Mahsa Arzani, Mohammad Ali Sahraian, Hamed Rezaei, Abdorreza Naser Moghadasi

**Affiliations:** 1 Department of Neurology, Sina Hospital, Tehran University of Medical Sciences, Tehran, Iran; 2 Multiple Sclerosis Research Center, Neuroscience Institute, Tehran University of Medical Sciences, Tehran, Iran; 3 Department of Urology, Sina Hospital, Tehran University of Medical Sciences, Tehran, Iran

**Keywords:** Chronic Recurrent Isolated Optic Neuritis, Recurrent Optic Neuritis, Neuromyelitis Optica Spectrum Disorder, Multiple Sclerosis

## Abstract

**Background:** Isolated relapsing optic neuropathy is a recurrent painful optic nerve inflammation without any sign of other demyelinating diseases such as multiple sclerosis (MS) or neuromyelitis optica (NMO) spectrum disorders, and the attacks are purely responsive to steroid therapy.

**Methods:** Recurrent isolated optic neuritis (RION) was diagnosed in patients who presented with at least two disseminating episodes of optic neuritis, and negative clinical, para-clinical, and radiological features of the demyelinating, infiltrative and vasculitis disorders involving optic nerve. The patients were assigned into two groups, chronic recurrent isolated optic neuritis (CRION) entailing patients with steroid dependent attack of optic neuritis and RION patients without steroid dependent attack of optic neuritis. They were monitored over a median of 4.0 ± 2.5 years.

**Results:** There were 16 women and six men with CRION and RION**; **with the median age of 31.7 ± 9.8 (29.3 ± 9.7 for women and 37.7 ± 7.7 for men). The women to men ratio was 2.6:1. The mean optic neuritis attack was 2.95 ± 1.32 in total. Eight patients were RION while 14 patients fulfilled CRION criteria and took long term immuno-suppressive drugs. In their follow-up, 4 out of 14 CRION cases (28.5%) showed clinical and concordant para-clinical features of NMO spectrum disorder. The analysis of demographic data showed that the average number of ON attacks in CRION patients (3.79 ± 2.32) was significantly more than the average in patients with RION (2.25 ± 0.46, P = 0.02).

**Conclusion:** CRION is a disease which requires aggressive glucocorticoid and long-term immunosuppressive therapy to restore visual acuity.

## Introduction

Recurrent optic neuritis could be a manifestation of different autoimmune diseases which involve central nervous system. Clinical as well as para-clinical hints may help to achieve the accurate final diagnosis. Recurrent optic nerve inflammation, at least in two disseminated episodes, without any evidence of systemic or central nervous system involvement, is called recurrent isolated optic neuritis (RION).^1^^-^^3^ If it is steroid dependent or requires continuous corticosteroid to prevent further attacks, it is diagnosed as chronic recurrent isolated optic neuritis (CRION).^4^^-^^7^ Steroid dependency is a crucial point in distinguishing CRION from RION.

A decade ago, Kidd, et al., described CRION syndrome as a unilateral or bilateral recurrent isolated optic neuropathy characterized by painful visual loss in association with inflammatory pathology.^8^ Moreover, according to their study, treatment with corticosteroid eliminates pain and improves visual acuity but steroid withdrawal causes tendency to relapse. Long term immunosuppressive therapy is recommended in most cases for preventing steroid side effects.^9^^-^^14^

The Patients with CRION and RION should neither have clinical nor para clinical features of demyelinating disorders such as multiple sclerosis (MS) and neuromyelitis optica (NMO), sarcoidosis and systemic autoimmune disease. Testing for anti-aquaporin 4 antibody (anti-NMO) can be helpful in distinguishing NMO spectrum disorders from CRION.^3^^,^^15^^-^^20^

The accurate diagnosis of optic neuropathy as CRION is important because its treatment, recovery and prognosis are different from those of RION, MS and other demyelinating disorders. Despite MS, optic neuritis resulting from CRION may lead to blindness or sever visual loss.^2^^,^^17^^,^^21^^,^^22^

The present study reports the demographic features and clinical characteristics of some Iranian patients with RION and CRION who referred to our center from different parts of the country.

## Materials and Methods

All patients presented with recurrent optic neuritis (at least two episodes) who referred to the MS Clinic at Sina Hospital, the major referral center for demyelinating disorders in Tehran, Iran, between 2003 and 2014, participated in the present study. The diagnosis of optic neuritis had been confirmed by an ophthalmologist as well as a neurologist. The patients were precisely assessed with lab tests and radiological evaluation to exclude those with demyelinating diseases of the central nervous system, and those with infiltrative, inflammatory, granulomatosis and vasculitis disorders involving the optic nerve.

Radiological studies were performed on the patients including orbital magnetic resonance imaging (MRI) and anterior optic pathway with fat suppression, brain, cervical and thoracic MRI and chest X-ray (for the detection of sarcoidosis). 

Patients without any evidence of specific lesion on brain and spinal MRI involvement were eligible to enter the study.

Blood sampling for complete blood count (CBC), electrolytes, thyroid function tests (TFT), erythrocyte sedimentation rate (ESR), C-reactive protein (CRP), antinuclear antibody (ANA), anti-double stranded DNA (anti dsDNA), vitamin B12, aquaporin 4-antibody (enzyme-linked immunosorbent assay method) and cerebrospinal fluid (CSF) sampling were obtained from all of the patients.

According to the data obtained from medical history, systemic and neurological examination, radiological, serological and spinal fluid investigation, only the patients with isolated idiopathic recurrent optic neuritis were included in this study.

Demographic findings of these cases, response to the treatment, and disease evolution during the survey were analyzed. The patients had some visits with every 6 months to 1 year interval. In addition, they were visited whenever required and after the attacks.

Recurrent attacks affecting the same eye were considered when there was at least 1-month interval between two attacks.

## Results

As indicated in the method, the patients diagnosed with a demyelinating, ophthalmologic, nutritional deficiency, infiltrating and inflammatory systemic disorders causing optic neuropathy, were excluded from the survey; thus only 16 women and 6 men had the criteria for CRION or RION.

Out of 22 patients, 14 clearly fulfilled the diagnostic criteria for CRION (corticosteroid dependency and attack recurring with steroid withdrawal) while the others were RION. 

The women to men ratio was 2.6:1. The mean age of the patients was 31.7 ± 9.8 years.

In the women group, the mean age was 29.3 ± 9.7 while it was 37.7 ± 7.7 for men, suggesting no significant statistical difference between the two groups (P = 0.07). The most prevalent age of onset was in the third and fourth decades of life with 54% and 27% for each decade respectively, with the age range of 9-45 years.

A total number of 54 attacks were recorded for all patients. The mean recurrence of attacks was 2.95 ± 1.32 (median 3) which showed no significant difference between the both genders (P = 0.54). 

**Table 1:** Demographic data of patients

**Total number**	**Female**	**Male**	**Total**	**P**
	**Mean ± SD (median)**	**Mean ± SD (median)**	**Mean ± SD (median)**	
	**16**	**6**	**22**	
Age at diagnosis (year)	29.3 ± 9.7 (29.0)	37.7 ± 7.7 (39.5)	31.7 ± 9.8 (31.0)	0.07
Age of onset (year)	26.4 ± 11.2 (26.8)	35.8 ± 7.2 (37.0)	29.1 ± 10.9 (29.9)	0.07
Number of attacks	3.06 ± 1.76	3.67 ± 2.65	2.95 ± 1.32 (3.0)	0.54
Duration of disease (year)	5.99 ± 3.60 (5.7)	6.91 ± 3.10 (6.0)	6.24 ± 3.43 (6.0)	0.57

SD: Standard deviation

P of < 0.05 are considered significant

The maximum number of episodes was 9 times in two patients; three patients experienced 4 attacks and it was less than 4 for the others. Four patients experienced bilateral simultaneous optic neuritis in one episode during the course of their disease. One patient had simultaneous bilateral optic neuritis at the onset and two further times during the follow-up. The demographic data is shown in table 1.

The demographic data is presented in detail for each group in table 2. Comparison of age of onset, duration of disease and follow-up between CRION and RION revealed no significant differences between the two groups, but the number of attacks was more in CRION before the beginning of treatment, compared to RION cases and the difference was significant (P = 0.02).

***MRI, CSF, and serologic test findings*** *: *The CSF analyses for cell count, protein and glucose were normal in all patients, and oligo clonal band (OCB) was negative in all patients except for one who showed just one unmatched band in CSF. 

Para clinical tests for vasculitis/autoimmune disorders were negative in all patients. 

Three patients had nonspecific T2 hyper intense lesions in white matter on brain MRI, the number of these T2 nonspecific brightness lesions remained unchanged during the brain MRI follow-up in the next 6 months and the following year. Spinal cord MRI was negative in all patients at the first evaluation.

NMO antibody was negative during the first evaluation in all patients.

***Clinical course of the disease:*** In the RION group, all patients responded completely to intravenous corticosteroid during their attacks and after tapering corticosteroid, they remained free of exacerbation for a mean time of two-year follow-up.

**Table 2:** Demographic data of each group

**Variable**	**CRION**	**RION**	**Total**	**P**
	**Mean ± SD**	**Mean ± SD**	**Mean ± SD**	
Age at diagnosis (year)	35.90 ± 3.40	34.00 ±6.60	31.70 ± 9.80	0.72
Male	44.70 ± 9.90	39.00 ± 9.90	37.70 ± 7.70	
Female	32.40 ± 13.40	32.00 ± 4.90	29.30 ± 9.70	
Age of onset (year)	29.30 ± 12.60	28.70 ± 7.20	29.10 ± 10.90	0.90
Male	37.00 ± 6.50	33.50 ± 10.60	35.80 ± 7.20	
Female	26.20 ± 13.40	26.70 ± 5.70	26.40 ± 11.20	
Duration of disease (year)	6.70 ± 4.07	5.43 ± 1.80	6.24 ± 3.43	0.55
Male	7.75 ± 3.59	5.25 ± 1.06	6.91 ± 3.10	
Female	6.29 ± 4.36	5.50 ± 2.07	5.99 ± 3.60	
Number of attacks	3.79 ± 2.32	2.25 ± 0.46	2.95 ± 1.32	0.02
Male	4.25 ± 3.20	2.50 ± 0.70	3.67 ± 2.65	
Female	3.60 ± 2.06	2.17 ± 0.40	3.06 ± 1.76	
Duration of follow up (year)	4.32 ± 2.91	3.35 ± 1.31	4.00 ± 2.54	0.54
Male	7.00 ± 3.55	4.75 ± 0.35	6.25 ± 2.99	
Female	3.25 ± 2.01	2.80 ± 1.09	3.10 ± 1.73	
Number of patients	14	8	22	
Male	4	2	6	
Female	10	6	16	

CRION: Chronic recurrent isolated optic neuritis; RION: Recurrent isolated optic neuritis; SD: Standard deviation;

P-value of < 0.05 is considered significant.

**Table 3:** Neuromyelitis optica spectrum disorder (NMOSD) cases

	**Case 1**	**Case 2**	**Case 3**	**Case 4**
Gender	Man	Man	Woman	Woman
Age of onset (year)	45	29	42	26
Duration disease (year)	13	5	8	4
Interval between disease onset and final diagnosis of NMOSD (year)	10	1	6	2
MRI positive findings	LETM	LETM	LETM	Periaqueductal hyperintensity
NMO antibody	Positive	Positive	Positive	Positive
Optic neuritis episodes	9	3	4	4

NMOSD: Neuromyelitis optica spectrum disorder; LETM: Longitudinally extensive transverse myelitis; MRI: Magnetic resonance imaging

In the CRION cases, two patients had one episode of optic neuritis who needed to be treated with plasma exchange in addition to the intravenous corticosteroid [one of them developed Neuromyelitis optica spectrum disorder (NMOSD) later]. After initiating the long-term immunosuppressive treatment (13 patients received azathioprine and only one patient received low dose prednisolone), patients were evaluated for their response to drug and disease status in regular visits of every 6 months.

Four out of fourteen CRION cases developed NMO spectrum disease during follow-up course (Table 3); three of them presented paraplegia and spinal cord syndromes, with extensive cord lesions compatible to NMO spectrum disorders. Anti-aquaporin 4 was positive in these three patients. In the other patient, follow-up MRI revealed periaqueductal hyper intensity in the brain and positive NMO antibody in the serum. 

The mean years between the onset of recurrent ON and final diagnosis was 4.75.

## Discussion

The prevalence of demyelinating disorders including MS is increasing. Although optic neuritis is one of the most common presenting symptoms in MS and NMO, there are still a small number of patients without any definite diagnosis even with the recurrence of the symptoms.^23^^-^^25^ RION and CRION are associated with those patients who manifest recurrent optic neuritis without a definite diagnosis of any other demyelinating diseases such as MS or NMO. They are more prevalent in women and may develop to other typical demyelinating diseases over time. Four of our cases developed NMOSD which is concordant with other studies.^20^^,^^26^^,^^27^ Except these converted cases, other CRION patients remained responsive to steroids and immunosuppressive therapy like the previous observations made on CRION.^6^^,^^11^ Only two of our patients had recurrent episodes with less favorable responses to treatment. 

Similar to the report presented here, other studies showed that the prevalence of CRION and RION is relatively higher among women.^1^^,^^2^^,^^11^^,^^22^^,^^28^ The strong point of this study is the comparison made between RION and CRION cases with respect to the age of onset, sex superiority, rate of attacks and disease conversion. Since the risk of blindness is high among CRION cases, recognition of this disease is of considerable importance. 

Other probable differential diagnoses were also made in the present study. In contrast to studies conducted by Myers, et al.^11^ and Lin, et al.^29^ there were not any evidence of vasculitis disorders or granulomatous diseases in the cases of the present study; moreover, no abnormality was found in the screening serologic test performed for autoimmune disorders, suggesting the presence of autoimmune optic neuropathy in the absence of systemic autoimmune process.^30^

Brain MRIs were normal except for three patients who had non-specific white matter lesions. This finding is compatible with the findings of other studies about brain MRI in CRION.^8^^,^^20^^,^^26^^,^^31^ Besides, except for one patient, OCB was negative.

Although the patients with NMOSD or MS were excluded before the beginning of the study, during the follow-up, some of the CRION cases converted to NMOSD. This conversion was also observed in other studies.^20^^,^^26^^,^^27^ Alongside the risk of conversion to MS and NMOSD, the higher rate of ON attacks and their severity in the CRION patients, predispose the optic nerve to an axonal damage. Optical coherence tomography (OCT) is a useful method to document this effect. With respect to two patients in CRION group, the optic neuritis attacks did not respond well to the treatment in the acute phase. As stated in the survey performed by Petzold and Plant, prognosis of recovery from optic neuritis is poorer among NMO and CRION cases compared to those with MS.^2^

The CRION cases in the present study (except for two cases who finally converted to NMOSD) were well controlled during the long term immunomodulatory treatment similar to the other studies.^6^^,^^11^ In addition to reports about azathioprine and methotrexate, cyclosporine, alkylating agents such as cyclophosphamide and chlorambucil, and intravenous immunoglobulin (IVIG) were among the choices of treatment.^11^^,^^14^^,^^22^ A case series of patients with RION reported failure of rituximab in controlling attacks in one patient, but a potent effect was reported for natalizumab on another patient.^32^ Thereafter, it can be concluded that due to the small data which is presented specially on more aggressive treatments, judgment about the choice of treatment is challenging.

The significant difference observed in the present study between CRION and RION in terms of means of ON attacks may be due to the higher number of attacks in CRION cases before the initiation of the therapy.

***Limitation:*** Unfortunately, there was no OCT data of the patients, which could add documentary information about optic nerve and retinal damage in both groups. The cell-based assay anti-NMO technique does not exist in Iran; therefore, we had to utilize enzyme-linked immunosorbent assay (ELISA) technique. It is hoped that future studies apply this novel method instead. 

Antibody against myelin oligodendrocyte glycoprotein is introduced as a diagnostic marker and can be used as a prognostic factor in patients with a seronegative anti-NMO, facilitating diagnosis of a subgroup of NMOSD. However, this method was not employed it in this study; it would be one of the prospective issues in further studies.

## Conclusion

Early immunomodulatory treatment is recommended to prevent further optic neuritis attacks and consequent axonal damage. Isolated optic neuritis may be dependent on steroids and should be followed and managed properly.

These patients may fulfill the criteria for NMO or MS, but most of them remain isolated even after several years. Early proper treatment with steroids or cytotoxic agents is recommended to prevent further optic neuritis attacks and consequent axonal damage.

## Notes:

**How to cite this article:** Arzani M, Sahraian MA, Rezaei H, Naser Moghadasi A. Recurrent isolated optic neuritis: A study on 22 patients. Iran J Neurol 2017; 16(3): 130-5.
